# Supplementary material for: MetaRibo-Seq measures translation in microbiomes
Source: Nat Commun. 2020 Jun 29;11:3268. doi: 10.1038/s41467-020-17081-z (PMC7324362; doi:10.1038/s41467-020-17081-z)
Supplement: Supplementary file 10 — Supplementary Data 7 [file 41467_2020_17081_MOESM10_ESM.zip › File2/Confidence_High_Taxonomy/291215_out.krona.html]

Javascript must be enabled to view this page.

members
magnitude
magnitudeUnassigned
count
unassigned
taxon
rank

291215\_out

88

88
superkingdom
2

976
phylum
88

88
class
200643

88
order
171549

171550
88
family

genus
16
239759
88

SRS012969\_contig\_number\_9825SRS023526\_contig\_number\_25918SRS047433\_contig\_number\_20299SRS062654\_contig\_number\_19947SRS076756\_contig\_number\_14459SRS077231\_contig\_number\_24989SRS077392\_contig\_number\_contig-100\_2962.238811SRS097958\_contig\_number\_6104SRS098644\_contig\_number\_44529SRS104036\_contig\_number\_7455SRS104400\_contig\_number\_53529SRS144297\_contig\_number\_13624SRS144714\_contig\_number\_15270SRS148319\_contig\_number\_15065SRS149181\_contig\_number\_20300SRS893259\_contig\_number\_2744

4
species

SRS045826\_contig\_number\_11629SRS075984\_contig\_number\_11018SRS077849\_contig\_number\_9738SRS149075\_contig\_number\_16107
1118061

1
species

SRS075878\_contig\_number\_2567
2364787

12
species

SRS011302\_contig\_number\_28787SRS015486\_contig\_number\_252SRS016203\_contig\_number\_11663SRS016629\_contig\_number\_123SRS045195\_contig\_number\_6537SRS046502\_contig\_number\_3336SRS075963\_contig\_number\_103SRS076804\_contig\_number\_13111SRS1041141\_contig\_number\_4881SRS148874\_contig\_number\_12568SRS893172\_contig\_number\_15436SRS893187\_contig\_number\_19399
1896970


SRS013940\_contig\_number\_21319SRS014235\_contig\_number\_39355SRS014923\_contig\_number\_46043SRS015264\_contig\_number\_21402SRS015579\_contig\_number\_22202SRS016335\_contig\_number\_13386SRS016989\_contig\_number\_2471SRS017307\_contig\_number\_15399SRS019445\_contig\_number\_2546SRS020328\_contig\_number\_34501SRS024009\_contig\_number\_8829SRS045004\_contig\_number\_12621SRS045645\_contig\_number\_24571SRS045713\_contig\_number\_18587SRS048060\_contig\_number\_contig-100\_3425.72480SRS063190\_contig\_number\_contig-100\_1989.92015SRS063985\_contig\_number\_15607SRS076976\_contig\_number\_16353SRS077024\_contig\_number\_9586SRS077641\_contig\_number\_23502SRS098061\_contig\_number\_contig-100\_3466.63838SRS1041092\_contig\_number\_10196SRS104311\_contig\_number\_19286SRS104975\_contig\_number\_21552SRS1054691\_contig\_number\_17062SRS1055022\_contig\_number\_25295SRS1055043\_contig\_number\_10776SRS1055067\_contig\_number\_contig-100\_919.103388SRS1055099\_contig\_number\_2234SRS142599\_contig\_number\_28623SRS142923\_contig\_number\_16188SRS143372\_contig\_number\_2437SRS148159\_contig\_number\_36492SRS149784\_contig\_number\_12675SRS893279\_contig\_number\_16317SRS893358\_contig\_number\_contig-100\_1056.66853SRS893378\_contig\_number\_9211
214856
37
species

species
1
626932

SRS077086\_contig\_number\_12597

species
17
1263035

SRS011405\_contig\_number\_contig-100\_1862.110767SRS018049\_contig\_number\_22SRS018936\_contig\_number\_12009SRS019496\_contig\_number\_11021SRS021484\_contig\_number\_20696SRS050299\_contig\_number\_14159SRS052078\_contig\_number\_3091SRS053214\_contig\_number\_3119SRS062701\_contig\_number\_6655SRS063489\_contig\_number\_7208SRS075821\_contig\_number\_13913SRS077194\_contig\_number\_5535SRS104084\_contig\_number\_5195SRS104693\_contig\_number\_10662SRS1055038\_contig\_number\_5505SRS1055069\_contig\_number\_15779SRS147766\_contig\_number\_19421
